# Prevalence and correlates of substance use among school-going adolescents (11-18years) in eight Sub-Saharan Africa countries

**DOI:** 10.1186/s13011-023-00542-1

**Published:** 2023-07-07

**Authors:** Nuworza Kugbey

**Affiliations:** School of Natural and Environmental Sciences, University of Environment and Sustainable Development, Somanya, Ghana

**Keywords:** Alcohol, Marijuana, Amphetamine, Adolescents, sub-Saharan Africa

## Abstract

**Background:**

Substance use constitutes a major public health issue especially among adolescents as it has associated adverse behavioural, health, social and economic outcomes. However, there is a paucity of comprehensive evidence on the prevalence and associated factors of substance use (alcohol, marijuana and amphetamine) among school-going adolescents in sub-Saharan Africa (SSA). This study examined the magnitude of substance use and its associated factors among school-going adolescents in eight eligible sub-Saharan Africa countries.

**Methods:**

Data for the study were obtained from the Global School-based Health Survey (2012–2017) of 8 countries in SSA (N = 16,318).

**Results:**

Findings showed overall prevalence rates of 11.3% (95%CI = 10.8 − 11.8%), 2% (95%CI = 1.8 − 2.2%) and 2.6% (95%CI = 2.3 − 2.9%) for current alcohol use, current marijuana use and lifetime amphetamine use, respectively between 2012 and 2017. Late adolescence (15–18 years), being male, anxiety, bullying, fighting, truancy, having close friends, current cigarette smoking and tobacco use are significant risk factors for alcohol use. Anxiety, truancy, current cigarette smoking, tobacco use and suicidal attempt are significant risk factors for marijuana use. Anxiety, bullying, truancy, current cigarette smoking, tobacco use and suicidal attempt are significant risk factors for amphetamine use. Parental knowledge of activity, supervision and respect of privacy are significant protective factors of substance use.

**Conclusion:**

There is the need for comprehensive public health policies beyond school-based psycho-behavioural interventions targeting the significant risk factors of substance use among school-going adolescents in SSA.

## Introduction

Substance use among adolescents is a major public health issue as its consequences transcend physical health, psychological problems and social problems to include truancy and poor academic performance due to memory problems [1–4]. According to the WHO, the adolescence period is a transition between childhood and adulthood, usually from 10 to 19years. Some early researchers have categorized adolescents between 10 and 14years as early adolescence and those between 15 and 19years as late adolescence [5]. The Global Burden of Diseases (GBD) study reported alcohol use to be the 4th leading cause of disability among youth between 10 and 24years with significant sex variations [6, 7]. Substance use among adolescents is associated with poor physical health outcomes, serious injuries, depression, anxiety, truancy, poor academic performance and other risky behaviours [1, 4, 5, 8–11].

Estimates from individual studies on alcohol use among in-school adolescents in SSA countries range from 10 to 44% [10, 12–14]. Apart from alcohol use, marijuana and amphetamine use are on the rise among adolescents with their associated negative consequences. Some multi-country studies have reported varying rates of substance use among in-school adolescents. For example, Peltzer and Pengpid [15] found 0.9% lifetime cannabis use and 1% lifetime amphetamine use among in-school adolescents from five Asian countries. However, individual country estimates of marijuana use among adolescents in sub-Saharan Africa range from 5 to 28% [16–18]. These high rates of marijuana use have been reported to have associated comorbid substance use and mental health problems.

Estimates of amphetamine use from individual countries in SSA range from 7 to 10% [1, 18]. However, the WHO African Region in 2021 asserts that “[a]mphetamine-type stimulants (ATS) such as ‘ecstasy’ and methamphetamine now rank as Africa’s second most widely abused drug type”. An earlier multi-country study conducted among in-school adolescents between 2009 and 2013 reported a 4.1% current marijuana and a 5.1% lifetime amphetamine use in nine sub-Saharan Africa countries [19].

Several risks and protective factors have been associated with substance use among adolescents especially in Africa. For example, socio-demographic characteristics such as grade in school, sex-being male, and age-older adolescents [20, 21], mental health-related factors such as depression, anxiety, suicidal behaviours and tobacco use [10], socio-environmental factors such as having experienced hunger, been bullied, having been in a physical fight and having been attacked [18, 20, 22] and parenting factors such as parental substance use, knowledge of activity, supervision and respect of privacy [18, 20, 23, 24] are implicated.

Apart from the study on cannabis and amphetamine use by school-going adolescents in nine SSA countries by Peltzer and Pengpid [19] which examined the Global School-based Health Survey (2009–2013), no recent multi-country studies have been conducted to (1) examine the burden of substance use (alcohol, marijuana and amphetamine) among adolescents using the most recent data from in-school adolescents in SSA (2012–2017) as well as (2) explore the risk and protective factors of substance use to inform targeted school-based and other public health interventions. The study by Peltzer and Pengpid [19] only examined cannabis and amphetamine without alcohol which has become a major public health issue among youth worldwide and in SSA in particular. This current study fills this gap by examining the prevalence and associated factors of substance use (alcohol, marijuana and amphetamine) among in-school adolescents in eight SSA countries to inform policy, practice and education.

## Methods

### Data and sample

Secondary data were used for this study. Data were obtained from the Global School-based Student Health Survey of 8 countries in SSA (N = 16,318) between 2012 and 2017 (see Table 1). The Global School-based Health Survey is sponsored by the World Health Organization (WHO) and the Centers for Disease Control and Prevention (CDC) to collect data on health behaviours and their associated factors in school-going adolescents across several low-income and middle-income countries. Health behaviours and related factors include alcohol use, dietary behaviours, drug use, hygiene, mental health, physical activity, protective factors, sexual behaviours, tobacco use, violence and unintentional injury. Data collection involved closed-ended questionnaires administered to in-school adolescents in the various countries. Multi-stage sampling technique was used and the eligible sample sizes from the eight countries are summarized in Table 1. For this study, 16,318 adolescents across the eight countries had the complete set of the study variables.

**Table 1:** Sample distribution of adolescents in the study

Countries	Data collection year	Sample(n)*	Percentage (%)
Benin	2016	1,584	9.71
Ghana	2012	2,428	14.88
Liberia	2017	1,275	7.81
Mauritius	2017	2,301	14.10
Mozambique	2015	1,163	7.13
Namibia	2013	3,072	18.83
Seychelles	2015	1,730	10.60
Tanzania	2014	2,765	16.94
Total	2012–2017	16,318	100.00

*= samples with complete cases of study variables

### Study variables

#### Outcome variables

There were three main outcome variables in this study (current alcohol use, current marijuana use and lifetime amphetamine use). Single items were used to measure each of the outcome variables. Current alcohol use was measured with the question “During the past 30 days, on how many days did you have at least one drink containing alcohol?” Responses ranged from 1 = 0 days to 7 = All 30 days. The responses were further recoded as 1 = 0 (No) and 2 to 7 = 1 (Yes). Current marijuana use was measured with the question “During the past 30 days, how many times have you used marijuana (also called dagga, weed, boom, cannabis, stop, grass, pipt, stop, and joint or other country-specific names)?” Responses ranged from 1 = 0 days to 7 = All 30 days. The responses were further recoded as 1 = 0 (No) and 2 to 7 = 1 (Yes). Lifetime amphetamine use was measured with the question “During your life, how many times have you used amphetamines or methamphetamines (also called tik, speed, bennies, uppers, black beauties, mollies, or splash, or other country-specific names)?” Responses ranged from 1 = 0 times to 5 = 20 or more times. The responses were further recoded as 1 = 0 (No) and 2 to 5 = 1 (Yes).

#### Explanatory variables

A set of explanatory variables including socio-demographic characteristics (age and sex), mental health variables (anxiety, loneliness and suicidal behaviours), socio-environmental factors (hunger, bullying, physical attack, fighting, tobacco use, cigarette smoking, truancy, and having close friends) and parental factors (supervision, connectedness, knowledge of activity and privacy) were used in the current study based on their relevance in influencing adolescents’ health-related behaviours [5, 9, 25, 26].

### Statistical analyses

**Fig. 1:**
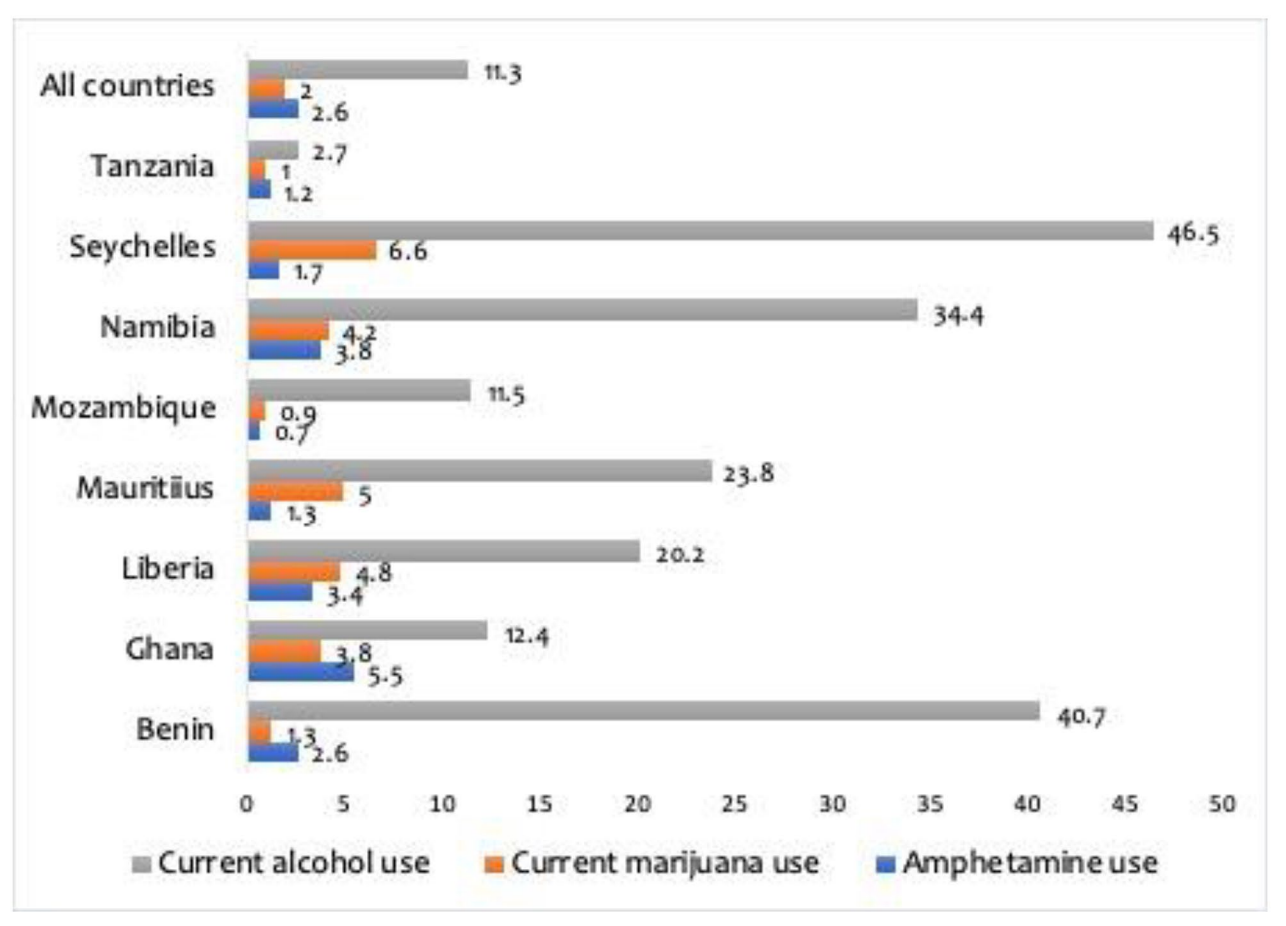
Prevalence of current alcohol use, current marijuana use, and amphetamine use

Stata Software version 17 (Stata Corporation, College Station, TX, USA) was used for the data analysis. Data from the eight countries were extracted from the WHO website, cleaned and recoded for the analysis. To pull all the data together, the append command was used to generate one dataset comprising the eight countries. Measurements of the prevalence of substance use (alcohol, marijuana and amphetamine) in the eight were done using counts and percentages with graphical illustration (Fig. 1). The bivariate associations between the explanatory variables and substance use were done using Pearson’s Chi-square test, and alpha level was set at 0.05. Multivariate analysis was done using logistic regression analysis with results presented in both unadjusted (OR) and adjusted (AOR) forms for each outcome variable. The odd ratios were presented with their 95% Confidence interval with statistical significance set at 0.05. Collinearity analysis was done and the results showed VIF values between 1.01 and 1.60 with a mean of 1.20. These results showed no evidence of substantial collinearity among the study variables. In all the analyses, the survey sampling weight was applied to ensure accurateness in the estimates from the surveys.

## Results

### Prevalence of substance use among adolescents in Africa

Results from Fig. 1 showed that the overall prevalence rates of substance use among school-going adolescents in SSA were 11.3%, 2% and 2.6% for current alcohol use, marijuana use and amphetamine use, respectively. Current alcohol use was highest in Seychelles (46.5%) and lowest in Tanzania (2.7%). Current marijuana use was highest in Seychelles (6.6%) and lowest in Mozambique (0.9%). The highest rate of lifetime amphetamine use was reported among adolescents in Ghana (5.5%), and the lowest rate of amphetamine use was reported among adolescents in Mozambique (0.7%).

### Associations between explanatory variables and adolescents’ substance use

Findings from Table 2 showed that all the explanatory variables in exception of respect of privacy (No privacy = 11.3% vs. Privacy = 11.1%, p = 0.165) were significantly associated with current alcohol use among the school-going adolescents. All the explanatory variables except having close friends (No = 2.8% vs. 1.9%, p = 0.493) were significantly associated with current marijuana use among school-going adolescents in SSA. It was further revealed that all the explanatory variables except social connectedness (No = 2.5% vs. Yes = 2.7%, p = 0.198) were significantly associated with current amphetamine use among school-going adolescents in SSA.

**Table 2:** Distribution of current alcohol, current marijuana, and amphetamine use across the explanatory variables

Variable	Weighted N	Weighted %	Current alcohol use	P-value	Current marijuana use	P-value	Amphetamine use	P-value
**Age group**				< 0.001		< 0.001		0.006
11-14years	6,821	41.8	6.0		1.9		2.6	
15-18years	9497	58.2	14.8		2.1		2.6	
**Sex**				0.002		< 0.001		< 0.001
Female	7,729	47.4	8.8		1.9		2.6	
Male	8,589	52.6	13.2		2.1		2.6	
**Felt hungry**				0.001		< 0.001		< 0.001
No	14,836	90.9	10.6		1.9		2.3	
Yes	1,482	9.1	15.8		3.7		5.6	
**Felt anxious**				< 0.001		< 0.001		< 0.001
No	14,850	91.0	10.0		1.6		2.1	
Yes	1,468	9.0	22.6		6.1		7.9	
**Felt lonely**				< 0.001		< 0.001		< 0.001
No	14,736	90.3	10.5		1.8		2.4	
Yes	1582	9.7	17.3		4.0		4.7	
**Bullied**				< 0.001		< 0.001		< 0.001
No	5,485	33.6	8.4		1.0		1.2	
Yes	10,833	66.4	16.6		4.1		5.5	
**Attacked**				0.028		< 0.001		< 0.001
No	9296	57.0	11.0		1.3		1.7	
Yes	7022	43.0	11.2		3.0		3.8	
**Engaged in fight**				< 0.001		< 0.001		< 0.001
No	11,651	71.4	9.3		1.0		1.6	
Yes	4667	28.6	15.7		4.6		5.1	
**Current cigarette smoking**				< 0.001		< 0.001		< 0.001
No	15,760	96.6	9.5		1.1		1.9	
Yes	558	3.4	56.2		28.1		23.3	
**Current tobacco use**				< 0.001		< 0.001		< 0.001
No	15,673	96.0	9.8		0.9		1.6	
Yes	645	4.0	44.0		28.3		27.6	
**Suicidal ideation**				< 0.001		< 0.001		< 0.001
No	14,082	86.3	10.2		1.4		2.0	
Yes	2,236	13.7	17.0		5.7		6.4	
**Suicidal plan**				< 0.001		< 0.001		< 0.001
No	14,169	86.8	10.1		1.4		2.0	
Yes	2149	13.2	17.7		6.1		6.5	
**Suicidal attempt**				< 0.001		< 0.001		< 0.001
No	14,180	86.9	9.8		1.1		1.6	
Yes	2138	13.1	20.2		5.8		9.2	
**Close friends**				< 0.001		0.493		0.010
No	1523	9.3	9.3		2.8		3.5	
Yes	14,795	90.7	11.3		1.9		2.5	
**Truant at school**				< 0.001		< 0.001		< 0.001
No	11,862	72.7	8.9		0.9		1.6	
Yes	4456	27.3	17.2		5.1		5.3	
**Supervision**				< 0.001		< 0.001		0.002
No	8,137	49.9	14.6		2.8		3.1	
Yes	8,181	50.1	7.7		1.2		2.1	
**Connectedness**				< 0.001		< 0.001		0.198
No	9778	59.9	12.5		2.5		2.5	
Yes	6540	40.1	9.1		1.3		2.7	
**Knowledge of activity**				< 0.001		< 0.001		< 0.001
No	9979	61.2	13.5		2.6		2.9	
Yes	6339	38.8	7.3		1.1		2.1	
**Privacy**				0.165		0.016		< 0.001
No	4570	28.0	11.3		3.1		4.5	
Yes	11,748	72.0	11.1		1.6		1.9	

*p-values were generated from the chi-square test

### Risk and protective factors of substance use among school-going adolescents

Results from Table 3 show that late adolescence (aOR = 2.63, 95%CI = 2.19–3.16), being male (aOR = 1.34, 95%CI = 1.16–1.54), anxiety (aOR = 1.86, 95%CI = 1.50–2.30), bullying (aOR = 1.49, 95%CI = 1.29–1.73), engaging in a fight (aOR = 1.32, 95%CI = 1.13–1.55), truancy (aOR = 1.49, 95%CI = 1.29–1.74), having close friends (aOR = 1.56, 95%CI = 1.22–2.00), current cigarette smoking (aOR = 5.41, 95%CI = 3.97–7.37) and tobacco use (aOR = 2.64, 95%CI = 1.93–3.59) significantly increased the odds for current alcohol use among school-going adolescents. However, parental supervision (aOR = 0.70, 95%CI = 0.60–0.82) and parental knowledge of activity (aOR = 0.63, 95%CI = 0.53–0.74) significantly decreased the odds for current alcohol use among school-going adolescents.

Anxiety (aOR = 1.90, 95%CI = 1.05–3.41), truancy (aOR = 3.20, 95%CI = 2.12–4.83), current cigarette smoking (aOR = 5.16, 95%CI = 3.03–8.79), tobacco use (aOR = 9.34, 95%CI = 5.62–15.52) and suicidal attempt (aOR = 2.21, 95%CI = 1.30–3.74] significantly increased the odds for marijuana use among school-going adolescents in SSA. However, parental knowledge of activity (aOR = 0.59, 95%CI = 0.36–0.96) and respect for privacy (aOR = 0.62, 95%CI = 0.40–0.97) significantly decreased the odds for marijuana use among school-going adolescents in SSA.

Anxiety (aOR = 2.16, 95%CI = 1.39–3.34), bullying (aOR = 2.25, 95%CI = 1.54–3.29), truancy (aOR = 1.83, 95%CI = 1.31–2.57), current cigarette smoking (aOR = 2.43, 95%CI = 1.42–4.17), tobacco use (aOR = 7.68, 95%CI = 4.91–12.02) and suicidal attempt (aOR = 1.86, 95%CI = 1.14–3.01) significantly increased the odds for amphetamine use among school-going adolescents in SSA. However, only parental respect of privacy (aOR = 0.52, 95%CI = 0.36–0.75) decreased the odds for current amphetamine use among school-going adolescents in SSA.

**Table 3:** Multivariable regression analysis of predictors or current alcohol use, current marijuana use, and amphetamine use

Variable	Current alcohol use	Current marijuana use	Amphetamine use
	aOR [95% CI]	aOR [95% CI]	aOR [95% CI]
**Age group**			
11-14years	1 [1.00,1.00]	1 [1.00,1.00]	1 [1.00,1.00]
15-18years	2.63^***^ [2.19,3.16]	0.83 [0.54,1.29]	0.75 [0.52,1.08]
**Sex**			
Female	1 [1.00,1.00]	1 [1.00,1.00]	1 [1.00,1.00]
Male	1.34^***^ [1.16,1.54]	1.08 [0.73,1.61]	1.02 [0.73,1.42]
**Felt hungry**			
No	1 [1.00,1.00]	1 [1.00,1.00]	1 [1.00,1.00]
Yes	1.09 [0.87,1.37]	0.85 [0.46,1.55]	1.28 [0.80,2.07]
**Felt anxious**			
No	1[1.00,1.00]	1 [1.00,1.00]	1 [1.00,1.00]
Yes	1.86^***^ [1.50,2.30]	1.90^*^ [1.05,3.41]	2.16^***^ [1.39,3.34]
**Bullied**			
No	1[1.00,1.00]	1 [1.00,1.00]	1 [1.00,1.00]
Yes	1.49^***^ [1.29,1.73]	1.36 [0.89,2.07]	2.25^***^ [1.54,3.29]
**Attacked**			
No	1 [1.00,1.00]	1 [1.00,1.00]	1 [1.00,1.00]
Yes	0.75^***^ [0.65,0.88]	0.94 [0.59,1.51]	1.11 [0.75,1.65]
**Engaged in fight**			
No	1[1.00,1.00]	1[1.00,1.00]	1 [1.00,1.00]
Yes	1.32^***^ [1.13,1.55]	1.39 [0.90,2.16]	1.09 [0.74,1.60]
**Truant at school**			
No	1 [1.00,1.00]	1 [1.00,1.00]	1 [1.00,1.00]
Yes	1.49^***^ [1.29,1.74]	3.20^***^ [2.12,4.83]	1.83^***^ [1.31,2.57]
**Felt lonely**			
No	1 [1.00,1.00]	1 [1.00,1.00]	1 [1.00,1.00]
Yes	1.03 [0.83,1.28]	0.88 [0.51,1.53]	0.77 [0.48,1.23]
**Current cigarette smoking**			
No	1 [1.00,1.00]	1 [1.00,1.00]	1 [1.00,1.00]
Yes	5.41^***^ [3.97,7.37]	5.16^***^ [3.03,8.79]	2.43^**^ [1.42,4.17]
**Current tobacco use**			
No	1 [1.00,1.00]	1 [1.00,1.00]	1[1.00,1.00]
Yes	2.64^***^ [1.93,3.59]	9.34^***^ [5.62,15.52]	7.68^***^ [4.91,12.02]
**Suicidal ideation**			
No	1[1.00,1.00]	1[1.00,1.00]	1 [1.00,1.00]
Yes	1.14 [0.92,1.43]	1.32 [0.78,2.23]	1.28 [0.80,2.07]
**Suicidal plan**			
No	1 [1.00,1.00]	1 [1.00,1.00]	1 [1.00,1.00]
Yes	1.10 [0.87,1.39]	1.59 [0.88,2.89]	1.26 [0.73,2.18]
**Suicidal attempt**			
No	1[1.00,1.00]	1[1.00,1.00]	1[1.00,1.00]
Yes	1.19 [0.95,1.49]	2.21^**^ [1.30,3.74]	1.86^*^ [1.14,3.01]
**Close friends**			
No	1[1.00,1.00]	1[1.00,1.00]	1 [1.00,1.00]
Yes	1.56^***^ [1.22,2.00]	1.53[0.87,2.71]	0.77 [0.45,1.33]
**Supervision**			
No	1[1.00,1.00]	1[1.00,1.00]	1 [1.00,1.00]
Yes	0.70^***^ [0.60,0.82]	0.75 [0.48,1.20]	0.94 [0.64,1.37]
**Connectedness**			
No	1[1.00,1.00]	1 [1.00,1.00]	1 [1.00,1.00]
Yes	0.99 [0.85,1.16]	0.77 [0.48,1.21]	1.63[0.57,1.24]
**Knowledge of activity**			
No	1 [1.00,1.00]	1[1.00,1.00]	1[1.00,1.00]
Yes	0.63^***^ [0.53,0.74]	0.59^*^ [0.36,0.96]	0.84 [0.57,1.24]
**Privacy**			
No	1 [1.00,1.00]	1[1.00,1.00]	1[1.00,1.00]
Yes	1.10[0.94,1.29]	0.62^*^[0.40,0.97]	0.52^***^ [0.36,0.75]
***N***	**16,318**	**16,318**	**16,318**
**pseudo** ***R*** ^**2**^	**0.148**	**0.384**	**0.261**

Exponentiated coefficients; 95% confidence intervals in brackets

^*^p < 0.05, ^**^p < 0.01, ^***^p < 0.001

## Discussion

Substance use predisposes adolescents to several physical, psychological [27], social and academic consequences. Understanding the burden of substance use in SSA is one of the major steps in addressing the menace with focus on key identified risk and protective factors. This study addressed this gap by examining the prevalence and the associated risk and protective factors of alcohol, marijuana and amphetamine use among school-going adolescents in SSA.

### Prevalence of alcohol, marijuana and amphetamine use among school-going adolescents

The overall prevalence rates of substance use among school-going adolescents in SSA were 11.3%, 2% and 2.6% for current alcohol use, current marijuana use and life time amphetamine use, respectively. The prevalence rates vary by country with school-going adolescents in Seychelles (46.5%) reporting the highest rate of current alcohol use and school-going adolescents in Tanzania (2.7%) reporting the lowest rate of current alcohol use. For current marijuana use, school-going adolescents in Seychelles (6.6%) reported the highest rate and school-going adolescents in Mozambique (0.9%) reported the lowest rate. The highest rate of lifetime amphetamine use was reported among school-going adolescents in Ghana (5.5%) and the lowest rate of amphetamine use was reported among adolescents in Mozambique (0.7%). These variations in the prevalence of substance use among school-going adolescents can be attributed to variations in the risk and protective factors of substance use within SSA countries. For example, multi-country studies in SSA have reported variations in risk factors for substance use such as bullying victimization [9], serious injuries [5, 26], truancy [25] and suicidal behaviours [19, 24]. Specifically, Seychelles has large tourist visits from western countries with liberal attitudes and practices regarding substance use including alcohol and marijuana which could influence school-going adolescents through observational learning. In the case of Ghana, the lack of effective drug enforcements could be one of the key contributing factors to high amphetamine use among in-school adolescents as some researchers have reported increasing use of drugs including tramadol [28]. One of the key contributing factors to these high rates and variations in substance use among adolescents in Africa could be lack of effective implementation of laws regarding substance use among underage youth in many African countries. Additionally, the easy access and availability of these substances as well as social norms [29] in the various countries might have contributed to the high rates of substance use among adolescents in Africa.

### Risk and protective factors of alcohol, marijuana and amphetamine use among school-going adolescents

Findings from the study showed late adolescence (15–18 years), being male, anxiety, bullying, fighting, truancy, having close friends, current cigarette smoking and tobacco use are significant risk factors for alcohol use. Several studies conducted in individual countries have found varied risk factors for alcohol consumption among in-school adolescents in Africa. Male adolescents are reported to engage in more risky behaviours than females [6, 30], and late adolescents have been noted for increased risky behaviours including alcohol consumption as reported in the GBD study on adolescents which found alcohol use to rank as the 3rd leading contributor to disease burden among late adolescent males [6]. The experience of mental health challenges and negative socio-environmental circumstances predisposes adolescents to the use of alcohol and other substances as a coping strategy to deal with their problems [10, 14].

Similar risk factors were found for marijuana and amphetamine use except for the socio-demographic characteristics. For example, anxiety, truancy, current cigarette smoking, tobacco use and suicidal attempt increased the risks for both marijuana use and amphetamine use. This is consistent with most of the country-level study findings [18, 31, 32] and multi-country-level findings among adolescents from the Caribbean, ASEAN and some African countries [15, 19, 33]. The experience of bullying was a significant risk factor for amphetamine use which is a cause for concern as bullying has been reported to be pervasive among adolescents in Africa [9, 34, 35]. The implication of these findings is that school-based intervention programmes aimed at addressing substance use among adolescents should take into consideration mental health and socio-environmental issues that predispose adolescents to engage in substance use behaviours.

Parental knowledge of activity, supervision and respect of privacy were found be significant protective factors of substance use. The role of parental involvement in adolescents’ risky behaviours has been widely reported by previous studies [10, 24, 30]. This is because when adolescents are monitored and given the necessary guidance in dealing with the myriad of challenges associated with the period of adolescence, they are less likely to engage in substance use behaviours. Thus, the role of parents in any intervention programmes aimed at addressing substance use should not be overlooked.

### Limitations

Although the cross-sectional study nature presents a major limitation to the findings, this study provides updated knowledge on substance use burden among in-school adolescents in SSA to inform adolescents health research, practice and policy. The different data collection years could serve as a limitation to the findings as these variations in the periods could influence the outcomes. For example, countries may have experienced different socio-economic or political environments which could have impacted on substance use among the adolescents. Due to the self-report nature of the data collection procedure, there could be social desirability biases which can influence the variations in the findings. It is also important to note that the prevalence of amphetamine cannot be compared with that of alcohol and marijuana since the periods of measurement differ, that is, lifetime prevalence for amphetamine and current prevalence (30days) for alcohol and marijuana. Despite these limitations, regular updates using most recent data from the Global School-based Student Health Survey is warranted. It is also important examine the trends in substance use across the various countries to understand the magnitude and patterns of substance use problems among adolescents in SSA. Wider policy-level factors were not examined in this study as the available data did not cover substance use policy variables.

## Conclusion

Substance use among in-school adolescents is a major public health issue, and the between country variations observed in this current study in the prevalence of alcohol, marijuana and amphetamine use suggests the need for country-specific programmes adapted to the needs and available resources within each country. Mental health and socio-environmental factors are significant risk factors of substance use in Africa, and urgent efforts are needed using a multi-sectoral approach to address this menace. High risks of alcohol use in adolescence could escalate into heavy alcohol use and alcohol dependence. Tobacco and alcohol use are ‘gateway’ substances that facilitate experimentation of marijuana and methamphetamine, and addressing these through public health policies could have an impact on other drug use among adolescents [36, 37]. Amphetamine, a relatively less used substance, is now becoming a common place among in-school adolescents in SSA and therefore requires concerted efforts to holistically address it.

## Data Availability

Data for this study can be obtained from https://extranet.who.int/ncdsmicrodata/index.php/catalog/GSHS.
